# Cutaneous eruption secondary to mothball inhalation

**DOI:** 10.1016/j.jdcr.2025.12.030

**Published:** 2025-12-29

**Authors:** Tessa M. LeWitt, Lily N. Clark

**Affiliations:** a Department of Dermatology, Columbia University, New York, New York; b Department of Dermatology, James J. Peters Veterans Affairs Medical Center, Bronx, New York

**Keywords:** cutaneous toxicity, ichthyosis, paradichlorobenzene, paraneoplastic acanthosis nigricans

## Introduction

Paradichlorobenzene (PDCB) is the active ingredient in common household deodorizers such as mothballs and toilet cakes. PDCB is toxic when ingested or inhaled; prolonged or repeated exposure may lead to a variety of cutaneous and systemic manifestations. In severe cases, PDCB toxicity can be permanent or fatal. In this report, we discuss a case of PDCB toxicity due to habitual inhalation of mothballs. This case underscores the importance of a complete review of systems, collateral history in the appropriate setting, and a basic understanding of PDCB toxicity.

## Case report

**Fig 1:**
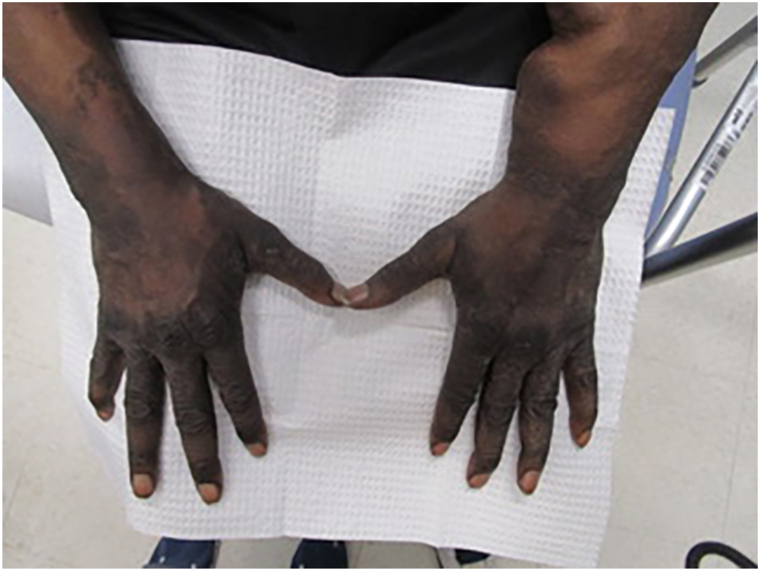
Brown ichthyotic plaques with overlying hyperkeratosis involving the dorsal hands and wrists.

**Fig 2:**
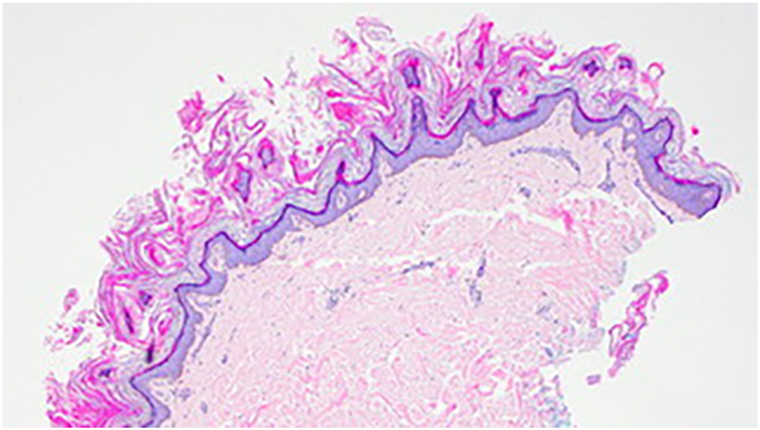
Punch biopsy revealed hyperkeratosis and papillomatosis with a sparse superficial dermal lymphocytic perivascular infiltrate.

A 62-year-old male with a medical history significant for congestive heart failure and end-stage renal disease was evaluated in our dermatology clinic for a 3-week history of rash on the hands. The rash was pruritic and had not responded to an empiric trial of nystatin cream prescribed by his primary care provider. He denied new exposures and had no prior dermatologic history. Review of systems was positive for an unintentional 10-pound weight loss over several months associated with worsening confusion and intermittent diplopia. Physical examination revealed brown ichthyotic plaques with overlying hyperkeratosis involving the dorsal hands and volar wrists (Fig 1). A punch biopsy revealed parakeratosis, papillomatosis, and spongiosis with a superficial dermal lymphocytic perivascular infiltrate; Periodic Acid-Schiff staining was negative for dermatophyte infection (Fig 2).

**Fig 3:**
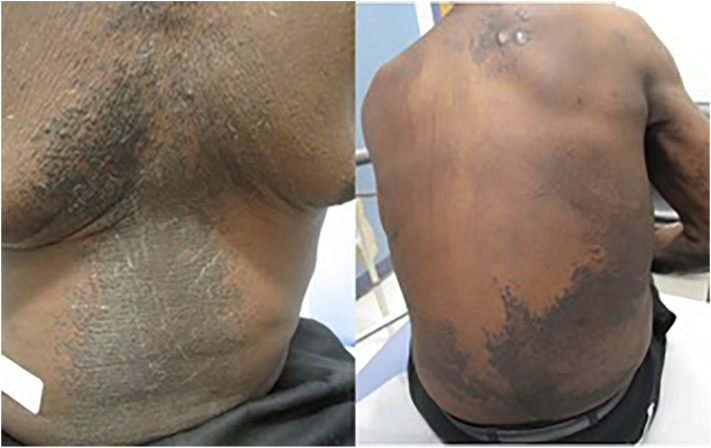
Velvety hyperpigmented plaques on the back (left), chest, and abdomen (right).

Our patient was treated symptomatically for pruritus with mid-potency topical steroids without benefit. He returned to clinic the following month with significant worsening of his rash. Physical examination at this time revealed velvety hyperpigmented plaques on the head and neck, trunk, and feet (Fig 3). There were also hyperpigmented ichthyotic plaques on the lower legs. At this time, he also reported worsening gait instability. Repeat biopsy at this visit showed hyperkeratosis and papillated epidermal hyperplasia consistent with acanthosis nigricans. His cutaneous findings in conjunction with unintentional weight loss and confusion raised initial suspicion for paraneoplastic acanthosis nigricans. However, extensive serologic and radiographic workup, including a complete blood count, complete metabolic panel, serum protein electrophoresis, myositis panel, colonoscopy, and computed tomography chest, was ultimately unrevealing.

During a follow-up visit, the patient was noted to smell strongly of mothballs. With his consent, collateral history was obtained from a present family member, who revealed that the patient had been inhaling mothballs for several years, with increasing frequency in recent months. The patient denied any ingestion. He was subsequently referred to psychiatry where he underwent cognitive behavioral therapy until he was able to discontinue the behavior. At a 3-month follow-up visit, his cutaneous and systemic symptoms had completely resolved.

## Discussion

Mothballs are common household deodorizers used to repel or kill moths and other insects that damage clothing and fabrics. Their active ingredient, PDCB, releases toxic vapors that deter or eliminate these insects. If stored improperly or misused, release of PDCB vapors from mothballs can pose serious health risks to humans. Exposure through inhalation or ingestion, particularly long-term exposure, has been associated with a variety of severe and life-threatening complications including encephalopathy and hemolytic anemia.^1^, ^2^, ^3^, ^4^, ^5^, ^6^, ^7^, ^8^ Proposed mechanism of toxicity is through lipophilic accumulation and generation of reactive oxygen species.

Some patients who present with PDCB toxicity report enjoying the smell of PDCB-containing products (ie, toilet cakes and mothballs), but there have also been reports of PDCB-associated pica and recreational drug use.^1^, ^2^, ^3^, ^4^, ^5^, ^6^ Many of these patients have an underlying psychiatric disease or substance abuse history.^1^, ^2^, ^3^, ^4^, ^5^, ^6^ Nonspecific systemic symptoms are often the first signs of toxicity, including anorexia, weight loss, malaise, and neurologic symptoms such as altered mental status, unsteady gait, diplopia, and urinary incontinence.^1^, ^2^, ^3^, ^4^, ^5^, ^6^ In severe cases, leukoencephalopathy related to PDCB toxicity can be detected with neuroimaging.^2^^,^^4^

Generalized rash is often described in association with PDCB toxicity but has only rarely been described morphologically and histologically. The eruption is characterized clinically by ichthyosiform, acanthosis nigricans-like, or lichenoid eruptions that are often generalized. Some patients report pruritus or pain.^1^^,^^4^^,^^5^ Histologically, skin biopsy may show parakeratosis, papillomatosis, and a sparse perivascular lymphocytic infiltrate.^1^^,^^4^^,^^5^

Management should begin with cessation of PDCB exposure, as most cutaneous and systemic symptoms will improve or resolve upon withdrawal. Suspected toxicity may be confirmed with urine testing for 2,5-dichlorophenol, a PDCB metabolite, although clinical history and physical examination are often sufficient to make the diagnosis. Basic laboratory work, including a complete blood count and a complete metabolic panel, should also be performed to evaluate for anemia, hepatotoxicity, and kidney injury. If a patient exhibits neurologic or psychiatric abnormalities, neuroimaging or lumbar puncture may be indicated. Treatment for the cutaneous manifestations is disappointing. A variety of skin-directed and systemic treatments have been trialed with minimal efficacy, including topical corticosteroids, emollients, phototherapy, and acitretin.^1^, ^2^, ^3^

Despite its rarity, dermatologists should be aware of this entity given its potentially life-threatening implications and reversible nature (Table I).

**Table I:** Patient demographics, comorbidities, and cutaneous and extra-cutaneous manifestations described in published case reports of PDCB toxicity

Case	1	2	3	4	5	6	7	8
PMID	30989101	23608871	22431793	31008163	16870927	16870927	24343157	33259053
Age/sex	50/F	40/F	48/F	19/F	18/F	18/F	Late 30/F	21/F
Comorbidities	Substance use disorder Depression Sickle cell trait Cirrhosis	Substance use disorder Depression Anxiety Hypertension	Diabetes mellitus Chronic kidney disease Chronic hepatitis C Cocaine abuse disorder	Bipolar disorder Anemia	NA	NA	Anemia Sickle cell trait Depression	Post-traumatic stress disorder Menorrhagia Iron deficiency anemia
Exposure	Ingestion, daily	Ingestion, 2 y	Ingestion	Inhalation, several months	Inhalation, daily for 10 minutes for 4-6 mo Chewing, half a mothball daily for 2 mo	Inhalation, 5-10 min daily for a few wk	Chewing, 1 bite per day several times per wk for >15 y	Ingestion, 2 y
Description of cutaneous manifestations	1-3 mm flat-topped violaceous papules coalescing into thin, confluent plaques	Dry, hyperpigmented rash	Xerosis, ichthyosiform scaling Diffuse lichenification, hyperpigmentation	Ichthyosiform hyperpigmented plaques	Ichthyosis-like dermatosis	Ichthyosis-like dermatosis	Ichthyosiform with prominent scaling	Ichthyotic papular eruption
Areas of involvement	Ears Upper extremities (wrists, forearms) Lower extremities (hip distal lower legs)	Ears Extremities (including hands) Trunk	Face Extremities (most pronounced on arms, hands) Trunk (upper)	Neck Trunk Extremities (including axillae)	Upper extremities (elbows, hands) Lower extremities	Upper extremities (elbows, hands) Lower extremities	Upper extremities (including hands) Lower extremities (including feet) Trunk (up to neckline)	Face Upper extremities Trunk Buttocks Lower extremities
Pruritus	Yes	Yes	Yes	Not reported	Not reported	Not reported	No	Yes
Extracutaneous manifestations	Neurologic: Encephalopathy Altered mental status Ophthalmologic: Blurry vision Systemic: Generalized weakness Fatigue Weight loss Failure to thrive Other: Pain in the extremities	Neurologic: Altered mental status Ataxic gait Cogwheel rigidity Slurred speech Somnolence Systemic: Anorexia	Neurologic: Altered mental status Somnolence Other: Hypotension	Neurologic: Altered mental status	Neurologic: Unsteady gait Urinary retention Mental sluggishness Cerebellar syndrome Hematologic: Iron deficiency Anemia Lymphoneutropenia	Neurologic: Unsteady gait	Neurologic: Leg weakness Truncal ataxia Altered mental status Bladder and bowel incontience Paresthesias Short-term memory loss Diminished strength Clonus Dysmetria Ophthalmologic: Blurred vision Opsoclonus Intranuclear ophthalmoplegia Nystagmus Systemic: Fatigue Anorexia	Neurologic: Falls Slurred speech Personality changes Impaired decision-making Mutism Opthalmologic: Blurred vision Systemic: Weakness Other: Malodor of axillae and oral cavity
Histopathology	(1) Mild acanthosis, alternating parakeratosis, perifollicular plugging (2) Thinning of the Malpighian layer, basket weave orthokeratosis, slight acanthosis with basal layer hyperpigmentation, and slight perivascular lymphocytic infiltrate	NA	NA	Papillomatosis, intact granular layer, and sparse perivascular lymphocytic infiltrate	NA	NA	NA	Papillated psoriasiform acanthosis with prominent parakeratosis and dyskeratosis within the superficial epidermis
Treatments	Hydrocortisone 1% cream Triamcinolone cream Emollients Acitretin 10 mg daily	Fluocinonide cream Hydroxyzine IV hydration Vitamin supplementation PEG tube feeding	Topical corticosteroids Emollients Antihistamines NBUVB Prednisone (5 mg every other day)	Supportive care	Not mentioned	Not mentioned	Interferon beta-1b Intravenous steroids Natalizumab Methylprednisolone (1 g daily × 5 d) Fluoxetine 60 mg daily	Not mentioned
Outcome	Complete resolution at 3 mo; relapse of habit	Recovered ability to talk, eat, and transfer after 4 wk	Death	Recovery of ability to speak and walk after several months	Complete recovery at 6 mo	Complete recovery at 3 wk	Ventilator dependent and minimally responsive, requires oxygen via tracheostomy	Death

*F*, Female; *IV*, intravenous; *NA*, not applicable; *NBUVB*, narrow band ultraviolet B; *PEG*, percutaneous endoscopic gastrostomy.

## Conflicts of interest

None disclosed.
